# Relating SARS-CoV-2 variants using cellular automata imaging

**DOI:** 10.1038/s41598-022-14404-6

**Published:** 2022-06-18

**Authors:** Luryane F. Souza, Tarcísio M. Rocha Filho, Marcelo A. Moret

**Affiliations:** 1 CCET, Universidade Federal do Oeste da Bahia, Barreiras, 47808-021 Brazil; 2 SENAI-CIMATEC, Salvador, 41650 -010 Brazil; 3 ICCMP & IF, Universidade de Brasília, Brasília, 70910-900 Brazil; 4 DCET, UNEB, Salvador, Brazil

**Keywords:** Evolution, Molecular biology, Physics

## Abstract

We classify the main variants of the SARS-CoV-2 virus representing a given biological sequence coded as a symbolic digital sequence and by its evolution by a cellular automata with a properly chosen rule. The spike protein, common to all variants of the SARS-CoV-2 virus, is then by the picture of the cellular automaton evolution yielding a visible representation of important features of the protein. We use information theory Hamming distance between different stages of the evolution of the cellular automaton for seven variants relative to the original Wuhan/China virus. We show that our approach allows to classify and group variants with common ancestors and same mutations. Although being a simpler method, it can be used as an alternative for building phylogenetic trees.

## Introduction

The disruption caused during the last two years by the COVID-19 pandemic is hard to be underestimated, from more than five million deaths and 270 million cases world-wide, according to official sources ^1^, to economic disruption in most countries^2^. In December 31, 2019 the first case was reported in the city of Wuhan, China, and in January 9, 2020, the World Health Organization (WHO) informed that Chinese scientists reported that the disease was caused by a new coronavirus. In February 11, 2020, in order to not associate the disease with any locality or groups of people the new coronavirus was named SARS-CoV-2 and the disease it caused COVID-19. In March 11 of that same year the WHO declared the outbreak a pandemic^3^.

The SARS-CoV-2 virus is part of the same virus family as the SARS-CoV and MERS-CoV viruses, the Sarbecovirus subgroup of the subdivision of the Betacoronavirus genera, which were responsible for epidemics in China (2003) and Saudi Arabia (2012)^4^. The last decade or so witnessed important developments in genome sequencing techniques, with a major increase in information gathering (data) on DNA, RNA, and protein sequences, as exemplified by the amount of data in databases such as GenBank^5^ and UniProt^6^ (for a more thorough account on genomic databases see^7,8^). Genomic information on animals, plants, and significant disease-causing viruses and bacteria are now easily available to researchers worldwide. Even before COVID-19 was declared a pandemic researchers in China determined the genomic sequencing of the virus^9^. Genomic sequencing is a crucial for designing vaccines, identify variants, determine the virus family and to drugs development^10–12^. The SARS-CoV-2 is a single-stranded RNA virus, with a genome size of 30 Kb, and four structural proteins: Nucleocapsid (N), Matrix (M), Envelope (E) and the Spike (S)^4,10^. The latter is responsible for recognizing and allowing the virus to enter the cell, possibly the main reason why this protein has been widely studied. Mutations in the SARS-CoV-2 viruses result in new variants with mutations in the spike protein increasing replication within cells, and an increased transmissibility^8^.

A protein can be depicted as a primary structure formed by a sequence of long strings of characters containing all information: structure, function, hydrophobicity and different motifs. Several researchers have studied how to extract different properties, e. g. hydrophobicity^13–16^, fractality^17,18^, geometric and thermodynamic aspects^19–21^. Cellular Automata have been widely used to model complex systems with simple, easy-to-understand rules^22^, and in recent years many papers were devoted study protein related problems using this approach. Sleit and Mdain^23^ proposed a protein folding model based on cellular automata, with straightforward evolutionary rules based on the hydrophobicity of amino acids. Other works dedicated to the same problem include^24–26^. Cellular Automata Image (CAI) analysis^27^ is a powerful tool to classify protein structure^28–30^ and virus taxonomy^31^. These images can contain important information on the modeled system, for example, CAI allows to differentiate similar systems with respect to those significantly different. The identification of functions, structures, location, and common ancestry of a protein sequence can be performed by a comparison with other know proteins in databases, using alignment, similarity, and homology techniques^32^. In the present paper we propose a protein comparison approach using a cellular automaton image and the information theoretic Hamming metric for the distance between such images, as a measure of similarity and difference, applied to the spike protein. The distance is measured with respect to the S protein in the initial virus strain as first detected in Wuhan, and for the following Variants Of Concern (VOCs) with mutations of the Spike protein: Alpha (first identified in the United Kingdom), Beta (South Africa), Gamma (Brazil), Delta (India), and the more recent Omicron (South Africa), B.1.1.28, and P2 (Brazil). Our goal is to explicitly obtain the evolutionary relationships between these SARS-CoV-2 variants.

The cellular automata image approach for protein classification and the Hamming distance are presented in “Methods” section. Our results are presented and discussed in “Results and discussion” section, and we close the paper with some concluding remarks in “Concluding remarks” section.

## Methods

Cellular automata are discrete dynamical systems with simple local evolution rules and, despite this, can show complex behavior^22^. The rules take into account the state of neighboring cells, analogous to protein structure since physicochemical characteristics of neighboring amino acids influence the folding or function of the protein. The cellular automata considered here has four components: a grid, the set of states, the neighborhood of each state, and the local transition rule. Several possibilities were proposed for encoding the sequence of the 20 types of amino acids in a protein: an 8-digit code for each amino acid^33^, or codes reflecting physicochemical characteristics and degeneracy, based on rules of similarity and complementarity: based on molecule recognition and information theory, with a 5-digit code for each amino acid^34^, or by representing the amino acid sequences using the hydrophobicity index of each amino acid^28^. The latter in the present work as it allows to better describe the evolutionary relationships between SARS-CoV-2 variants, resulting in smaller distantes for variants with the same mutations and those that emerged in the same period throughout the pandemic. It also groups together variants that share a mutation in the amino acid N501Y. Coronaviruses that cause MERS, SARS and COVID-19 diseases are all closely related, and it is natural to expect that the same coding scheme will be a good representation of the SARS-CoV-2 proteins based in the same molecules. This is reinforced by the discussion in^35^ (see particularly Figure 3 of this paper) that shows that the Spike proteins of these viruses have very similar characteristics. Different binary codes were used to distinguish SARS-CoV viruses from other coronaviruses, such as the one used by Xiao et al.^34^, which is a simpler code and does not take into account physicochemical amino acids.

**Figure 1:**
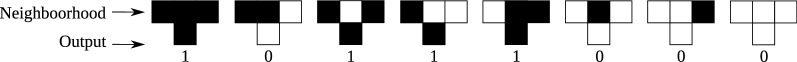
Rule 184 from^36^ for an elementary cell automaton with three neighbors. The state 0 is represented in white and 1 in black.

Table 1 shows the coding of Ref.^28^ that will be used throughout the rest of this work. The Spike protein sequence has 1273 amino acids, and each one is coded as a 5 digit sequence, and thence $N=6365$ cells with 0 and 1 as possible state, and composing the first line of the cellular automata (initial condition). The state of the *i*-th cell at step *t* is notated as $x_i^t=0,1$, *i*, $i=1,\ldots ,N$. The neighborhood of the cell at position *i* is composed by the three cells at positions $i-1$, *i* and $i+1$, resulting in $2^3=8$ different states for the neighborhood. We also use periodic boundary conditions. For each possible configuration of the neighborhood, the middle cell can assume two possible states, and thus the number of possible evolution rules is given by $2^8=256$^36^. As discussed in^36^ and ^31^, the most appropriate evolution for the celular atomaton rule for SARS-CoV virus classification and for distinguishing them from other viruses, is Wolfram’s 184 and depicted in Fig. 1. This rule yield as a typical feature of SARS-CoV viruses a V pattern pattern in the cellular automaton image (see below).

**Table 1:** Coding for each of the 20 possible amino acids^28^.

Amino acids	K	N	D	E	P	Q	R
Decimal code	6	8	9	10	11	12	13
Binary code	00110	01000	01001	01010	01011	01100	01101

Amino acids	S	T	G	A	H	W	Y
Decimal code	14	15	16	17	18	20	21
Binary code	01110	01111	10000	10001	10010	10100	10101

Amino acids	F	L	M	I	V	C	
Decimal code	23	24	26	27	28	30	
Binary code	10111	11000	11010	11011	11100	11110	

In order to implement a numeric metric to distinguish different images, we consider here the information theoretic Hamming distance $D_H$, which is commonly used for the distance between sequences of same length and is a simple measure the number of different positions/errors with all required mathematical properties^37^. Here the sequences considered are the states of the automata at the same step*t*. In this case the distance can be written as:

1 $$\begin{aligned} D_H(t)= \frac{1}{N} \sum _{i=1}^{N}\Vert x_i^t-\overline{x}_i^t\Vert , \end{aligned}$$

with *N* the size of the grid, $x_i^t$ the state of the cell at step *t* for the S protein in the initial Wuhan strain and $\overline{x}_i^t$ the Spike protein of the given variant.

### Ethics declarations

No human samples/human data were used in the present work.

## Results and discussion

**Figure 2:**
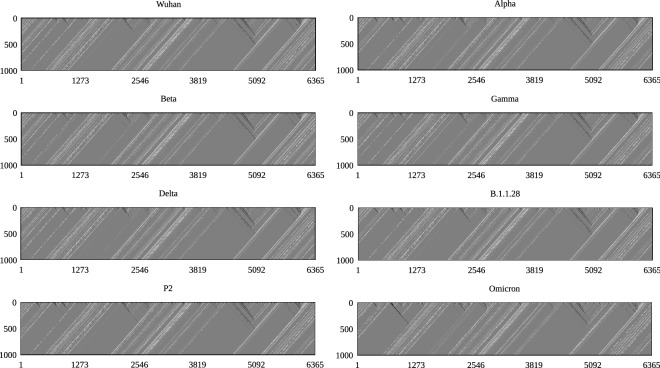
Evolution of protein cellular automata from coding in from Table 1 and the Wolfram’s rule in Fig. 1, for the different variants. The horizontyal and vertical axes are the cell number *i* and the evolution step *t*, respectively.

**Figure 3:**
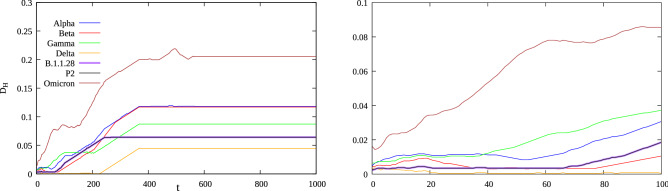
Left: Hamming distance as a function of step *t* for the time evolution of the cellular automata associated to the spike protein between each variant and the initial Wuhan strain. Right: Zoom over the initial values of *t*.

The cellular automaton for the SARS-CoV-2 spike protein using available genomic data data for Alpha, Beta, Gamma, Delta, B.1.1.28, P2 variant and the original strain are available at^5^ and^38^ for Omicron, represented with the coding in Table 1, and evolved according to the rule in Fig. 1 over 1000 time steps. Deletions in the protein sequence were represented by the code 00000 and insertions by introducing the deletion code in the other proteins at the corresponding position. Figure 2 shows the resulting image representing the evolution of the automaton for each considered variant, where the *V* shaped patterns characteristic of SARS-CoV viruses^31^ are clearly visible. Figure 3 shows the time evolution of the Hamming distance $D_H$ for each variant with respect to the original Wuhan strain. For the initial steps the distance has small values, as expect for variants of the same virus, and increases with *t* up to an asymptotic constant value after approximately $t=400$ steps. The small number of mutations, if compared to the number of amino-acids in the protein and measured by the small Hamming distance at $t=0$, is amplified by the evolution of the cellular automata and results in quite different asymptotic values of $D_H$, after an irregular transient of roughly 200 time steps. This allows us to classify the cellular automata as Wolfram Class IV, with an intermediate behavior between Classes II (periodical) and III (chaotic). Although the Omicron variant presents more mutations (and therefore a higher value of $D_H$) than other known VOCs, with 33 amino acid changes in the spike protein^39^, its distance plot remains close to the variants sharing the N501Y mutation (see Table 2 for the characteristic mutations of each variant). This large number of modifications seems to be linked to an increased transmissibility and possibly smaller efficiency of curent vaccines^40^.

Table 2 shows the different mutations present in each main variant of the SARS-CoV-2 virus. We then see from Fig. 3 that the present approach groups the variants carrying the N501Y mutation, the sense that final stationary Hamming distance between these variants and the original are more closer and with higher values. The Gamma and P2 variants are also closer as they have the same clade B.1.1.28 (note that the distance for P2 and B.1.1.28 are practically the same in the Figure), while the Delta variant, which carries the P681R mutation unfamiliar to the other variants, is the one with smallest distance. We believe that the present approach is a straightforward way to measure evolutionary distances between SARS-CoV-2 variants, much simpler that other techniques as in^41,42^ were a normalized Laplacian pyramid is employed to measure pairwise similarities in cellular automata image wavelet images in order to build phylogenetic trees.

**Figure 4:**
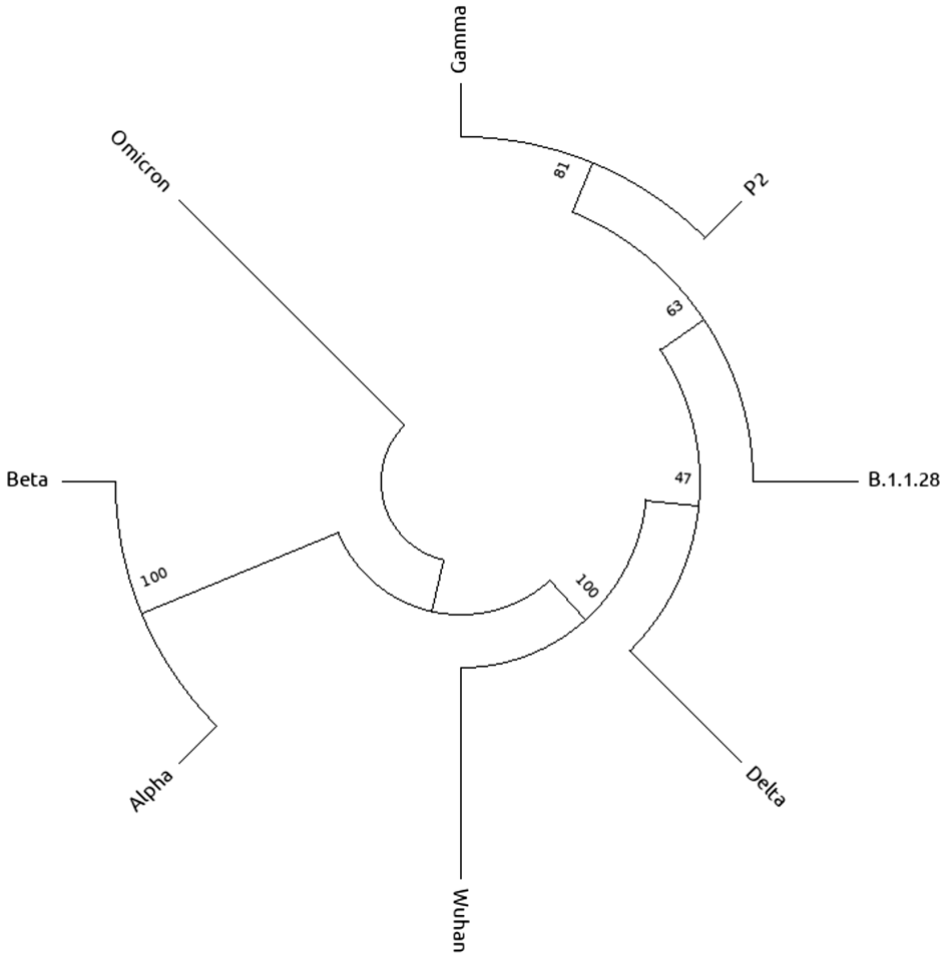
Phylogenetic tree of SARS-CoV-2 variants and the Wuhan strain sequences from the neighbor-joining method^43^.

In order to show that the present approach properly relates the variants, we computed the phylogenetic tree from the the neighbor-joining method with alignment^43^, which calculates evolutionary distances between species. Figure 4 shows the results for the main known variants of SARS-CoV-2. We see that the variants Gamma, P2, and B.1.1.28 are in the same clade in the tree, while in Fig. 3 these same variants have closer stationary distances. Our results for the Hamming distance fo Delta, Gamma, P2, and B.1.1.28 variants shows that they are closer to the protein initially found in the Wuhan strain, as expected as they are in the same clade in Fig. 4. The same occurs for Alpha and Beta variants, which are in the same clade and have close stationary Hamming distances, while in both approaches the Omicron variant is clearly separated from the other variants. On the other hand, variants B.1.1.28 and P2 have the same stationary Hamming distance, as they have very similar mutations (see Table 2) while P2 is more close to Gamma in the phylogenetic tree.

**Table 2:** Mutations the Spike protein of the SARS-CoV-2 variants from^39^.

Variant	Mutations
Alpha	HV69-70del, Y145del, N501Y, A570D, D614G, P681H, T716I, S982A, D1118H
Beta	L18F, D80A, D215G, R246I, K417N, E484K, N501Y, D614G, A701V
Gamma	L18F, T20N, P26S, HV69-70del, D138Y, Y145H, R190S, K417T, E484K, N501Y, D614G, H655Y, T1027I, V1176F
Delta	T95I, G142D, E154K, L452R, E484Q, D614G, P681R, Q1071H
B.1.1.28	HV69-70del, Y145del, D614G, V1176F
P2	HV69-70del, Y145del, E484K,D614G,V1176F
Omicron	A67V, HV69-70del, T95I, G142D, VYY143-145del, N211I, L212del, G339D, S371L, S373P, S375F, K417N, N440K, G446S, S477N, T478K, E484A, Q493R, G496S, Q498R, N501Y, Y505H, T547K, D614G, H655Y, N679K, P681H, N764K, D796Y, N856K, Q954H, N969K, L981F

The variants with smallest values of $D_H$ are those with the smallest number of mutations in Table 2: Delta, B.1.1.28 and P2, which are also the variants without the N501Y mutation. Despite the differences in the images of each variant, resulting from different mutations, the celular automaton rule also results in the V-shaped pattern for SARS-CoV-2 type coronaviruses. This V pattern is characteristic of SARS-CoV-like coronaviruses as discussed in length in Refs.^31,36^. Despite the fact that the SARS-CoV-2 virus is different from SARS-CoV, they share this pattern from their common ancestors. During the COVID-19 pandemic many mutations occurred in the virus sequence, but without a functional change in the Spike protein, although some of these mutations may bring some advantages. However, since different sequencesi perform the same function, mutations in proteins are degenerate, a behavior fundamental for natural selection to occur. Without degeneracy, there is no genetic variability, and this hinders natural selection from acting^44^.

## Concluding remarks

The approach presented here allows to cluster variants with common ancestors by using a cellular automaton and the asymptotic Hamming distance for the resulting images for each variant, as shown in Fig. 2, and is a more straightforward and simpler evolutionary classification of those variants, than other approaches such as alignment technique, similarity analysis and image processing. Iti particularly discerns the deviation of Omicron with respect to other variants, preserving the V shaped pattern characteristic of the SARS-CoV viruses, despite having the largest number of mutations among known variants, and grouping variants with the N501Y mutation. Furthermore, after just three iterations of the automaton for the protein in the Wuhan strain, the amino acid at position 501 changed from N to Y. This rapid convergence suggest an alternative explanation for the emergence of Alpha, Beta, and Gamma on three continents simultaneously, an evolutionary convergence. We also note that without degeneration, mutations could lead to unfavorable structures for the virus, making it easier to control its spread^44^. Cellular automata are a simple tool to extract meaningful information from proteins sequences, with a very low computational cost. We hope that the present work will contribute as an useful tool to build protein phylogenetic trees.

## Data Availability

Datasets used during the current study are the sequences of the Spike proteins of the virus initially found in Wuhan [YP_009724390.1] and its variants Alpha [QWP89177.1], Beta [UAL50115.1], Gamma [QXF22923.1 ], Delta [QXP08802.1], Omicron [UGO97992.1], B.1.1.28 [QQK84800.1] and P2 [QXF22396.1] which are available at https://www.ncbi.nlm.nih.gov/genbank/.
